# Testing Empirical Support for Evolutionary Models that Root the Tree of Life

**DOI:** 10.1007/s00239-019-09891-7

**Published:** 2019-03-18

**Authors:** Derek Caetano-Anollés, Arshan Nasir, Kyung Mo Kim, Gustavo Caetano-Anollés

**Affiliations:** 1 Department of Evolutionary Genetics, Max-Planck-Institut für Evolutionsbiologie, Plön, Germany; 2 Department of Biosciences, COMSATS University, Islamabad, 45550 Pakistan; 3 Division of Polar Life Sciences, Korea Polar Research Institute, Incheon, Republic of Korea; 4 Evolutionary Bioinformatics Laboratory, Department of Crop Sciences, and Illinois Informatics Institute, University of Illinois at Urbana-Champaign, Urbana, IL 61801 USA

**Keywords:** Phylogenetic analysis, Superkingdoms, Fold superfamily, Tree of life, Evolution, Characters

## Abstract

Trees of life (ToLs) can only be rooted with direct methods that seek optimization of character state information in ingroup taxa. This involves optimizing phylogenetic tree, model and data in an exercise of reciprocal illumination. Rooted ToLs have been built from a census of protein structural domains in proteomes using two kinds of models. Fully-reversible models use standard-ordered (additive) characters and Wagner parsimony to generate unrooted trees of proteomes that are then rooted with Weston’s generality criterion. Non-reversible models directly build rooted trees with unordered characters and asymmetric stepmatrices of transformation costs that penalize gain over loss of domains. Here, we test the empirical support for the evolutionary models with character state reconstruction methods using two published proteomic datasets. We show that the reversible models match reconstructed frequencies of character change and are faithful to the distribution of serial homologies in trees. In contrast, the non-reversible models go counter to trends in the data they must explain, attracting organisms with large proteomes to the base of the rooted trees while violating the triangle inequality of distances. This can lead to serious reconstruction inconsistencies that show model inadequacy. Our study highlights the aprioristic perils of disposing of countering evidence in natural history reconstruction.

**Electronic supplementary material:**

The online version of this article (10.1007/s00239-019-09891-7) contains supplementary material, which is available to authorized users.

## Introduction

Phylogenetic characters are useful biological features. They carry history when they spread in evolution as they transform from a character state to another. Trees of life (ToLs) use a tree paradigm to describe the history of the diversified world, even if this history is network-like (Caetano-Anollés et al. 2018). Trees must be reconstructed from useful characters that change at rates appropriate to the evolutionary depth of the recovered trees capturing vertical phylogenetic signatures. However, not many characters are sufficiently conserved, especially characters describing macromolecular sequences. Caetano-Anollés and Caetano-Anollés (2003) published the first rooted genomic ToL reconstructed from a census of protein-structural domain in proteomes. Structural domains show distinct 3-dimensional compact fold structures that are both highly conserved and recurrent in proteomes (Murzin et al. 1995). They can be efficiently identified using hidden Markov models of structural recognition (Gough et al. 2001). Some proteomes may hold one such domain defined at some level of protein classification while others may hold many. For example, domains belonging to the TIM beta/alpha barrel superfamily that is common in enzymes can be found in the hundreds in a proteome. We have shown that domains are excellent phylogenetic characters because they are highly conserved (Nasir and Caetano-Anollés 2012). Their numbers in a proteome (genomic abundance) define character states, which embody ‘serial homologies’ and can be used to build and root ToLs (reviewed in Caetano-Anollés et al. 2014, 2018). Note that domain gain and loss establish proteome history when changes in domain abundance (character state transformations) are reconstructed by optimization along the branches of a tree (e.g. Nasir et al. 2014).

ToL reconstructions require designing character-specific state transformations models that are somehow faithful to the biological process of change. As we will describe, the model of Caetano-Anollés and Caetano-Anollés (CA^2^) uses standard additive-ordered (Wagner) characters that are fully reversible (Caetano-Anollés and Caetano-Anollés 2003) to build unrooted trees (e.g. Nasir et al. 2015). These trees are then rooted a posteriori with Weston’s generality criterion, which also embodies the method of Schwartz and Dayhoff of rooting with paralogous genes (reviewed in Caetano-Anollés et al. 2018). In sharp contrast, Harish and Kurland (HK) proposed a non-reversible model that uses unordered characters and a direct Sankoff optimization method to root ToLs (Harish et al. 2013; Harish and Kurland 2017a). The HK model has been criticized for being unrealistic and self-inconsistent (Kim et al. 2014; Nasir et al. 2017). It penalizes the growth of domain abundance and diversity in the protein world and violates the triangle inequality, a property that ensures that the minimum distance (cost) between any two points (states) is a straight line. Here, we evaluate the empirical support for evolutionary models that root ToLs reconstructed from domain counts in proteomes. We do so by comparing the performance and fit of the CA^2^ and HK models to the data using two published proteomic datasets.

## Materials and Methods

We tested two published datasets to evaluate the reliability and realistic nature of phylogenetic models. The proteome dataset from Harish et al. (2013) included abundance information for 1732 fold superfamily (FSF) structural domains in 141 cellular proteomes. We labeled this dataset the *HK dataset* since HK used it to establish their model. The dataset from Kim and Caetano-Anollés (2011) included abundance information for 1420 FSFs in 102 proteomes, all from organisms with free-living lifestyles. We labeled this dataset the *CA*^*2*^*dataset* since it was implemented with the CA^2^ model for comparison with the HK model (Kim et al. 2014). FSFs were defined by the Structural Classification of Proteins (SCOP) database (Murzin et al. 1995; Andreeva et al. 2008). FSFs are groups of structural domains that are evolutionarily conserved and unified by common descent. In both datasets, proteomes were equally sampled from the three superkingdoms Archaea, Bacteria and Eukarya.

Raw proteomic abundance values for each FSF were normalized, rescaled, and coded into 32 character states using an alphanumeric scale (0–9 and A–V) and phylogenetic matrices presented in Nexus file format. Maximum parsimony was used to search for the most parsimonious trees using the phylogenetic software PAUP* ver. 4.0a (build 157; August 2017)(Swofford 2003). Custom models were defined in the Nexus ‘assumptions’ block using the DefType, UserType, TypeSets and Ancstates commands. Custom asymmetric stepmatrices automatically rooted the phylogenetic trees. Similarly, transformation types derived from reconstructed frequencies of character change also rooted phylogenies intrinsically. Weston’s generality criterion implemented using the Lundberg rooting method (Lundberg 1972) placed the root at the most parsimonious location. Character state reconstructions (CSR) were implemented using Mesquite ver. 3.2 (Maddison and Maddison 2017) and MacClade ver. 4.08 (Maddison and Maddison 2002). Bubble charts were used to describe the frequency of unambiguous changes between states in FSF abundance. Tree topologies were compared with tanglegrams and tree distances using Dendroscope ver. 3 (Huson and Scornavaca 2012).

## Results and Discussion

### Order and Direction in Character Evolution

**Fig. 1:**
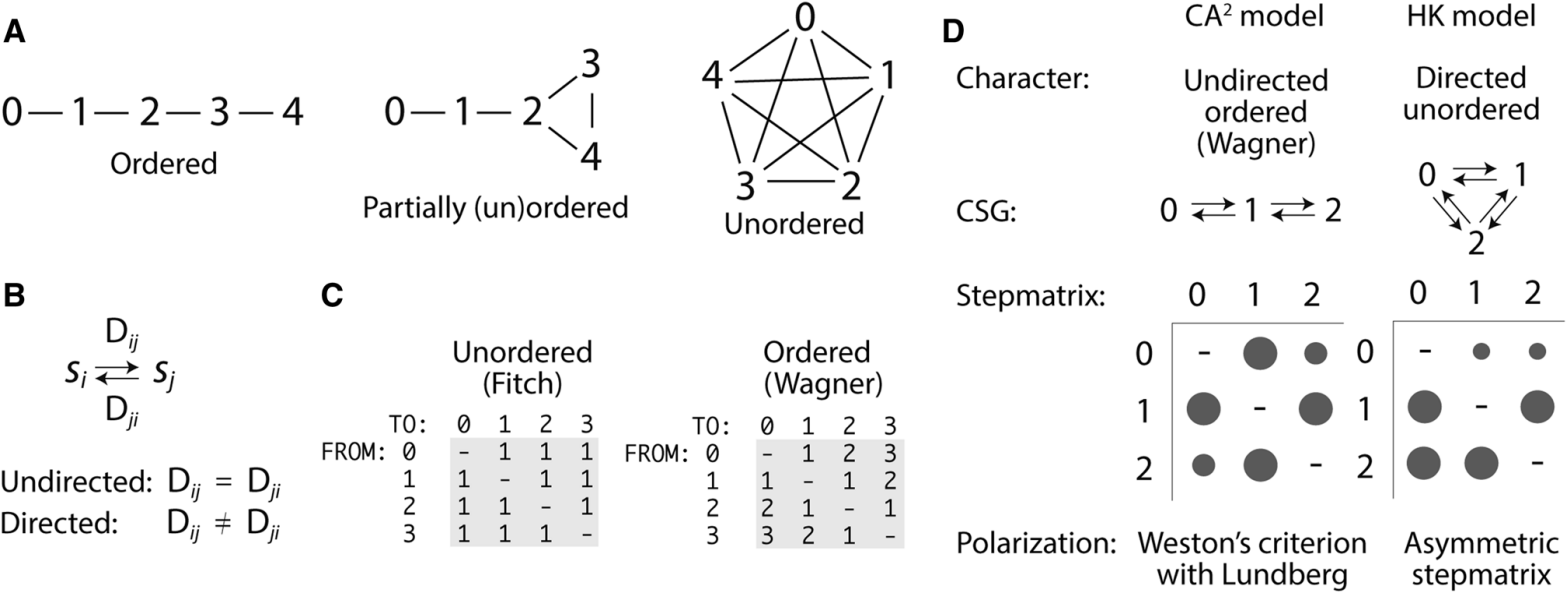
Evolutionary models in parsimony analysis. **a** Character state graphs (CSGs) for equally weighted undirected characters with five character states (0, 1, 2, 3 and 4). The CSG in the left is a typical maximally connected character, an ‘unordered’ character while the CSG in the right is a minimally connected character embodied in a ‘fully ordered’ character. The CSG in the middle is a partially ordered CSG containing a reticulation. **b** Transformation between character states can be undirected or directed depending on the costs D*ij* applied to the transformation from character state *i* to state *j*, or vice versa, with *i* ≠ *j*. **c** Character state matrices (stepmatrices) describing ordered and unordered character transformations. The matrices show state indices describing transformation costs (in tree lengths) from one character state to another. **d** Phylogenetic models discussed in this manuscript that are relevant to proteome evolution. Models are described by the undirected or directed characters they use, their CSGs, the stepmatrices describing probabilities of transformation between character states, and the polarization strategy that is used to root the resulting phylogenetic trees. The diameter of bubbles in stepmatrix diagrams is proportional to probabilities of character state change

Since not all character states must necessarily transform into each other in a character state transformation model (also known as a *transformation series*), character state graphs (CSGs) have been used to describe landscapes of possible evolutionary change (Slowinski 1993). CSGs connect *n* vertices representing character states with edges representing allowed state transformations (Fig. 1a). At one end of the spectrum of possible CSGs, maximally connected CSGs are ‘complete graphs’ that allow all possible transformations between states. Characters of this kind were first proposed by Kluge and Farris (1969) but their optimization was elaborated by Fitch (1971). They are commonly known as *unordered characters* or Fitch characters and are very popular when analyzing molecular sequences of proteins and nucleic acids. At the other end of the spectrum, minimally connected CSGs are non-reticulated graphs with *n*–1 edge connections. The simplest of these CSGs are linear CSGs with no vertices having three or more connecting edges. Their transformation series can be represented with alphanumeric strings, which makes them amenable for simple computation. Linearly connected characters were originally formalized by Kluge and Farris (1969) and optimized by Farris (1970). They are known as *ordered characters* or Wagner characters and are widely used in the analysis of serial homologies, especially those describing morphological features of organisms. A recent empirical comparison of ordered and unordered methods using inter-tree retention indices (Grand et al. 2013) supports the early conclusion of Slowinski (1993) that ordered characters are much superior in terms of both increasing resolving power and diminishing resolution artefacts. These studies revealed that ordered characters should be especially preferred when characters are quantitative and states describe gradients or clines (e.g. domain abundance counts).

Transformation changes define distances that are usually measured in steps. When characters are *undirected*, the number of steps between any two character states in one direction must match the number of steps in the opposite direction. In contrast, when they are *directed*, the number of steps can be different (Fig. 1b). This difference imposes directionality (a ‘polarization’) to character transformation and defines a flow of change in the direction of time. Stepmatrices are usually used to make transformation costs explicit during dynamic programming algorithmic implementations (Sankoff and Rousseau 1975). These square matrices list the ‘cost’ in number of steps of all possible transformations. Note that transformations requiring many steps are costlier from a parsimony point of view and are, therefore, less probable from an evolutionary point of view. Thus, cost and probability are inversely related. Figure 1c shows typical stepmatrix examples using transformation costs. In Fitch parsimony, change occurs between any state of an unordered character, with a cost of only one step, regardless of the direction of change. Thus, all probabilities of change between character states are equal in its corresponding stepmatrix. In Wagner parsimony, change between any two states of a linearly ordered transformation carries a cost equal to the number of edges separating the states, regardless of the direction of change. For example, the distances between states 0 and 2 or states 2 and 0 in the ordered CSG of Fig. 1c involve 2 steps while those between 0 and 4 or 4 and 0 involve 4 steps. While changes in Wagner characters are additive and more restrictive, both Fitch and Wagner characters are considered typical undirected characters that are fully reversible. Their associated stepmatrices are symmetric and their phylogenetic optimization by minimization of independent origins (homoplasy) produces unrooted phylogenetic trees, in which no ‘arrow of time’ is defined.

To root trees and convey history, a branch holding the ancestor of each optimal unrooted tree must be identified a posteriori and ‘pulled down’ to its base to reorient change along branches, doing so most-parsimoniously and in the direction of time. Wagner trees build from typical Wagner-ordered undirected characters can be rooted with direct or indirect rooting methods. Direct methods use character information present in the ingroup (the set of taxa being studied) to root the trees using, for example, the ontogenetic or generality criterion (Bryant 1997). In contrast, indirect methods root the trees by defining outlying groups of taxa (outgroups) as being ancestral. Because ToLs cannot be rooted with outgroups, phylogenomic trees of proteomes and domains built from structural domain counts in proteomes (Caetano-Anollés and Caetano-Anollés 2003) are rooted with direct rooting methods such as Weston’s generality criterion implemented empirically with Lundberg (Lundberg 1972)(see explanation below). These CA^2^ models are summarized in Fig. 1d. Alternatively, trees can be rooted by invoking character polarization directly in the model. In contrast with the unordered and ordered ‘static’ characters described by symmetric stepmatrices (e.g. Fitch and Wagner models), characters with transformations described by asymmetric stepmatrices produce rooted trees directly during Sankoff tree optimization. For example, the HK model (Fig. 1d) uses unordered characters to define a maximally connected CSG and assigns arbitrary probabilities to sets of character transformations (Harish et al. 2013). The asymmetric matrices tax gains more than losses, which forces ‘backward’ character state polarization and rooting of trees. This results in ‘upside down’ phylogenies that attract organisms with large proteomes to the base of the rooted trees (Kim et al. 2014). The HK model is also at odds with considerable background knowledge, including the scale-free property of domain networks, genomic scaling laws, inferences from the reconstruction of ancestors, and the fossil record (Kim et al. 2014; Nasir et al. 2017). Furthermore, since probabilities of change between states in CSGs depend on their direction, the HK model resembles parametric models in which each probability of the stepmatrix must be ‘pre-specified’ as a parameter of the model. This fact is important because pre-specification of each one of these parameters in the HK model results in many additional ad hoc assumptions.

### Rooting Trees a Posteriori with the CA^2^ Model

The relationship between the states of a CSG and the polarity of change are distinct concepts, regardless of the level of CSG connectivity (Mabee 1989). Evolution unfolds in time and is, therefore, an asymmetric ‘directional’ process. Thus, establishing the direction of character transformation along branches of a CSG is a necessary step that must be defined at some point in the course of phylogenetic analysis. Operationally, this ‘polarization’ can be done a priori by making assumptions of character polarity before searching for optimal rooted trees (e.g. directed characters of the HK method), or a posteriori once optimal unrooted trees have been identified without polarization (e.g. rooting of trees generated with undirected characters in the CA^2^ method). In other words, a priori polarization defines assumptions of polarity prior to tree optimization while a posteriori polarization defines assumptions once trees have been already recovered. Both a priori and a posteriori approaches will produce the same outcome in the absence of character conflict in phylogenetic data, i.e. in the absence of ad hoc hypotheses of homoplasy. However, data are never conflict-free and the purpose of phylogenetic analysis is to resolve it (Farris 1983). For that reason, Meacham (1984) suggested that characters be treated first as undirected and that the resulting unrooted trees be rooted with exploratory polarization methods and explicit parsimony arguments. This has been the popular view of modern phylogenetic analysis. We note that polarizing a CSG results in a rooted tree, and vice versa, rooting a tree results in CSG polarization.

The CA^2^ model takes full advantage of the benefits of Wagner characters and a posteriori polarization. The CA^2^ method generates optimal unrooted trees from multistate ordered characters with Wagner optimization and then applies the Lundberg optimization strategy to attach a hypothetical ancestor most parsimoniously to the internode of the unrooted trees recovered during tree optimization (Lundberg 1972). Polarization is done a posteriori. It is not done a priori by “pre-specification” of an ancestor as wrongfully claimed (Harish and Kurland 2017a); see Nasir et al. (2017) for extensive discussion. The ‘standard’ implementation of Lundberg sets all character states of the ancestor as ‘missing’ using the ‘ancstates’ command (ancstate = ?) and proceeds to optimize attachment of all possible ancestors to optimal trees, a strategy that complies with Weston’s rule and the generality criterion (Bryant 1997). Alternatively, arbitrarily defined ancestors can be optimally attached to the most parsimonious tree reconstructions. These ancestors delimit alternative Lundberg polarization schemes that can be compared with the ‘standard’ implementation to determine if some schemes are more parsimonious than others and are less affected by homoplasy. We have repeatedly observed in ToL reconstructions generated for over a decade that most parsimonious ToLs generated with the ‘standard’ and ‘all-0’ ancestor implementations of Lundberg are not only topologically isomorphic but are also optimal in terms of tree length and ensemble retention indices (RI). Tree length evaluates the most parsimonious solutions of Lundberg. RI is a measure of tree support that tests both the fit of character data to a reconstructed tree and levels of homoplasy in the analysis (Farris 1989). In all cases, optimally rooted ToLs consistently supported the Archaea-first hypothesis (Caetano-Anollés et al. 2014), in sharp contrast to ToLs build with the HK model, which are rooted in the branch leading to Eukarya.

**Fig. 2:**
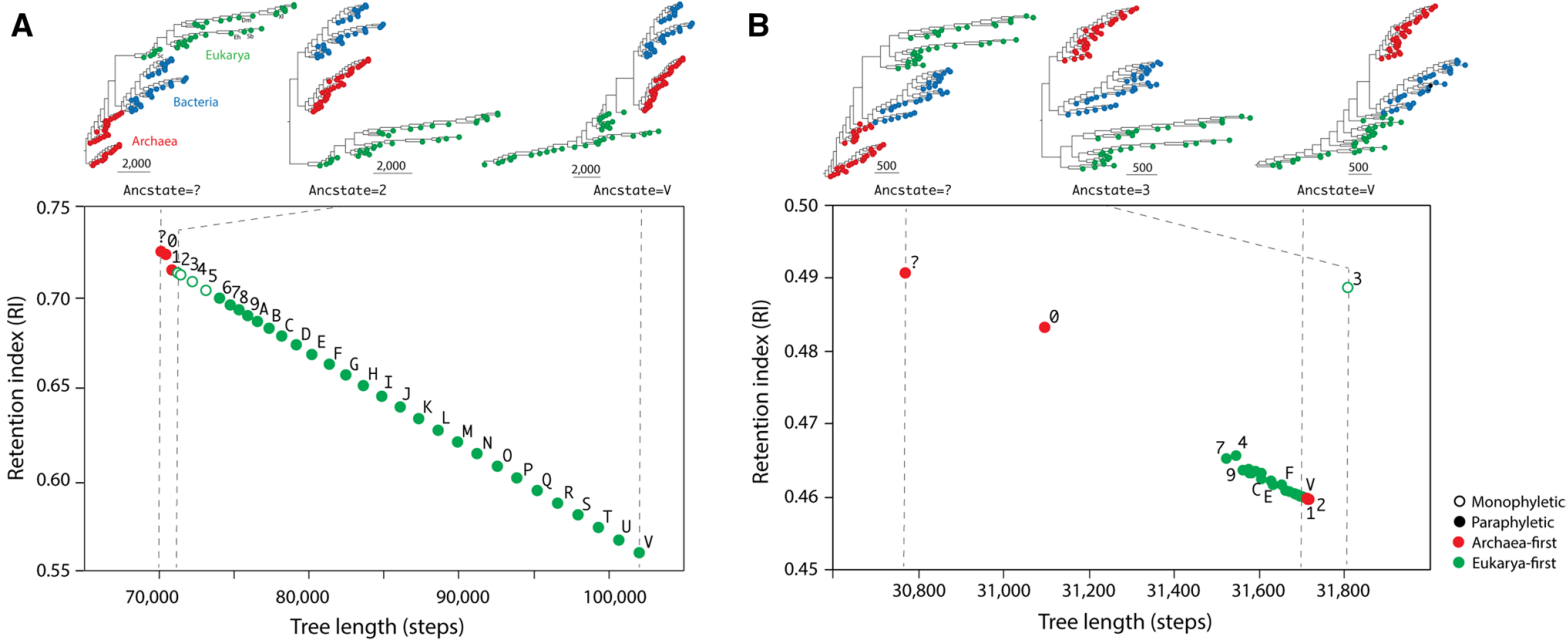
Rooting trees of life (ToLs) a posteriori with the Lundberg method. ToLs were generated using ordered (**a**) and unordered characters (**b**) from phylogenetic encodings of a genomic census of 1420 fold superfamilies (FSFs) of protein structural domains in 102 proteomes (*CA*^*2*^*dataset*). Proteomes were from organisms with free-living lifestyles belonging to superkingdoms Archaea (red), Bacteria (blue) and Eukarya (green). Ensemble retention indices (RI) of recovered trees were plotted against tree length. Topologies with paraphyletic or monophyletic basal superkingdoms were labeled with open and close circles, respectively, and colors describing support for an Archaea-first or Eukarya-first evolutionary scenario of origin. Sc, *Saccharomyces cerevisiae*; Eh, *Emiliania huxleyii*; Sb, *Sorghum bicolor*; Dm, *Drosophila melanogaster*; Xl, *Xenopus laevis*

To illustrate the analytical benefits of a posteriori polarization, we used the undirected ordered characters of the CA^2^ model to analyze the effect of alternative polarization scenarios in the reconstruction of ToLs generated from proteomic counts of structural domains in the *CA*^*2*^*dataset* (Fig. 2a). The ‘standard’ implementation of Lundberg sets all character states of the ancestor as ‘missing’ using the ‘ancstates’ command (ancstate = ?) and proceeds to optimize attachment of all possible ancestors to optimal unrooted trees, a strategy that complies with Weston’s rule and the generality criterion (Bryant 1997). Alternatively, arbitrarily defined ancestors can be optimally attached to the most parsimonious tree reconstructions. These ancestors delimit alternative Lundberg polarization schemes that can be compared with the ‘standard’ implementation to determine if some schemes are more parsimonious than others and are less affected by homoplasy. Figure 2 plots RI values against the length of the most parsimonious trees when these were rooted with ancestors holding the same ancestral state for every character. All 32 possible states in the ordered series (labeled in alphanumeric format from 0 to 9 and A to V) were explored as being ancestral. An RI value of 1 implies perfect fit and absence of ad hoc assumptions of homoplasy. An RI value of 0 implies the tree fits data as poorly as possible and exhibits maximum instances of independent origin. The ‘standard’ Lundberg implementation and Lundberg with ‘all-0’ and ‘all-1’ ancestors (i.e. ancestors that assign state 0 or state 1 to the entire character ensemble) were the best out of all possible implementations. They produced most parsimonious rooted ToLs with identical topologies that were the shortest, had the highest RI values, and placed Archaea at their base. This topological isomorphy and optimality of ToL reconstructions using the ‘standard’ and ‘all-0’ ancestor implementations of Lundberg have been repeatedly observed in our laboratories for over a decade. Thus, maximum parsimony consistently supports the Archaea-first hypothesis (Caetano-Anollés et al. 2014). Results have two important implications for phylogenetic analysis of proteomes: (i) the rooting scheme complies with Weston’s rule and the generality criterion, the rooting method that uses the least number of ad hoc assumptions (Bryant 2001), and (ii) optimal character polarization with ‘all-0’ ancestors shows there is a tendency of growth of structural domains in proteomes, and not global tendencies of reductive evolution as HK suggest. Results, therefore, add to the long list of evidence in support of the evolutionary principle of continuity (Nasir et al. 2017).

One remarkable finding is that RI and tree length decrease monotonically with perfect fit (*y* = − 5.10^− 6^ × + 1.07; *R*^2^ = 1.000; *P* < 0.001) when character states of Lundberg ancestors were defined with increasing values (from ancstate = 0 to ancstate = V). This regular monotonic decrease that manifests when polarization of the ordered series is gradually ‘inverted’ provides strong indication that proteome abundance data significantly fits the CA^2^ model of ordered characters and evolutionary growth in the nested lineages of the ToL. In fact, the regular patterns suggest the nesting of serial homologies is equally affected by increasing suboptimality of the rooting variants. As a control, we analyzed the effect of alternative Lundberg polarization scenarios on ToLs generated using unordered maximally connected characters (Fig. 2b). Again, the Lundberg ‘standard’ and ‘all-0’ ancestors were the most parsimonious and produced trees rooted paraphyletically in Archaea. However, there was no monotonic decrease pattern since the relationship between RI and length was haphazardous. The fit of abundance data to model was weaker.

### Evaluating the Biological Realism and Difficulties of the HK Model

Maximally connected characters are evolutionarily the most agnostic and least informative of all possible models. Mickevich labeled them “nihilistic” in that they are maximally agnostic and unrealistic (Mickevich 1982). HK chose a maximally connected CSG as foundation for their HK model. They then took a small fraction of possible character state transformations in the maximally connected CSG (~ 3% of the 992 possible transformations) and endowed them with a same but different probability of change, penalizing structural domain growth in proteomes. For their binary data matrices of domain occurrence, gains were taxed two times more than losses. For multistate data matrices of domain abundance, gains were taxed three times more than losses for the first gain. Stepmatrix penalties were chosen based on the premise that *“the propensity for change in superfamily frequency due to innovation is more constrained than the changes in frequencies (copy number) due to duplication and loss of members of a superfamily”* (Harish and Kurland 2017a). Crucially, the penalty value for innovation (twice for occurrence and thrice for abundance) was chosen as the “smallest penalty for each taxon sampling that supports a fully resolved tree with monophyletic clades that is consistent with the major groups identified in the corresponding unrooted trees”. The “major groups” that must be monophyletic in the HK model were defined by Harish et al. (2013) as superkingdoms Archaea, Bacteria and Eukarya. Thus, the *‘*empirical’ support for the HK model rests on an assumption that arises from the reconstructed phylogenetic tree (the end product of the phylogenetic exercise) and not the data or model that is used to build the historical hypothesis. This is troublesome for several reasons.

First, adding an assumption of monophyly amounts to adding an additional ad hoc *hypothesis* that is external to phylogenetic analysis with the sole purpose of disposing conflicting observations of independent origins. This additional hypothesis can constrain or override the minimization of ad hoc hypotheses of homoplasy that occurs during tree optimization. Within the phylogenetic hypothetico-deductive framework, severity of test decreases when invoking *ad hocs* and auxiliaries, often resulting in a “verificationist slippery slope [that] ultimately ends in tautology” (Kluge 1997). Typical examples of such ad hocs and auxiliaries invoked in the HK model include a priori weighting of characters or character transformation costs compatible with some form of phylogenetic congruence (such as monophyly-driven penalties), considering assumptions of pattern and process (such as incorporation of arguments on superfamily innovation), or adding an assumption of rooting (such as penalties in asymmetric stepmatrices). These difficulties are exacerbated when making global statements typical of a ToL.

Second, hypotheses of evolutionary pattern must be ‘discovered’ and must not be ‘pre-specified’ in phylogenetic analysis. HK claim penalties must be derived from “Hennig’s premise … that all species arise through the nested bifurcations that describe phylogenetic divergence from a common ancestor” (Harish and Kurland 2017a). However, by definition, all species of a ToL arise from a common ancestor even in the absence of monophyly in some region of the tree, so the foundational premise of the argument is faulty.

Third, monophyly is a relative statement that depends on the rooting of a tree and the definition of ingroups. Thus, manipulating character polarization to root a tree or invoking background knowledge to define an ingroup defeats the phylogenetic enterprise. Since the HK model forces monophyly of superkingdoms, the superkingdom-related ingroups constrain rooting to only three branches out of hundreds. However, there is no guarantee that ingroups have been properly defined or that further exploration will fine grain superkingdoms into smaller groups.

### Testing Empirical Support for the CA^2^ and HK Models

Since their introduction by Dayhoff et al. (1978), amino acid substitution matrices are widely used models for the evolutionary analysis of amino acid sequences. PAM and BLOSUM matrices describe amino acid change in sequence alignment data assumed to represent the extant world of protein sequences. This change is derived from amino acid mutability and frequency in sequences. Thus, stepmatrix substitution models can be directly derived from phylogenetic data. This can be done within the generalized maximum parsimony framework. Given a phylogeny and an initial evolutionary hypothesis one can reconstruct histories of character state change along the branches of the optimal trees and use this information to define a model of character evolution (Maddison and Maddison 2002). Doing so could be useful to test how compatible is an evolutionary model to actual data.

**Fig. 3:**
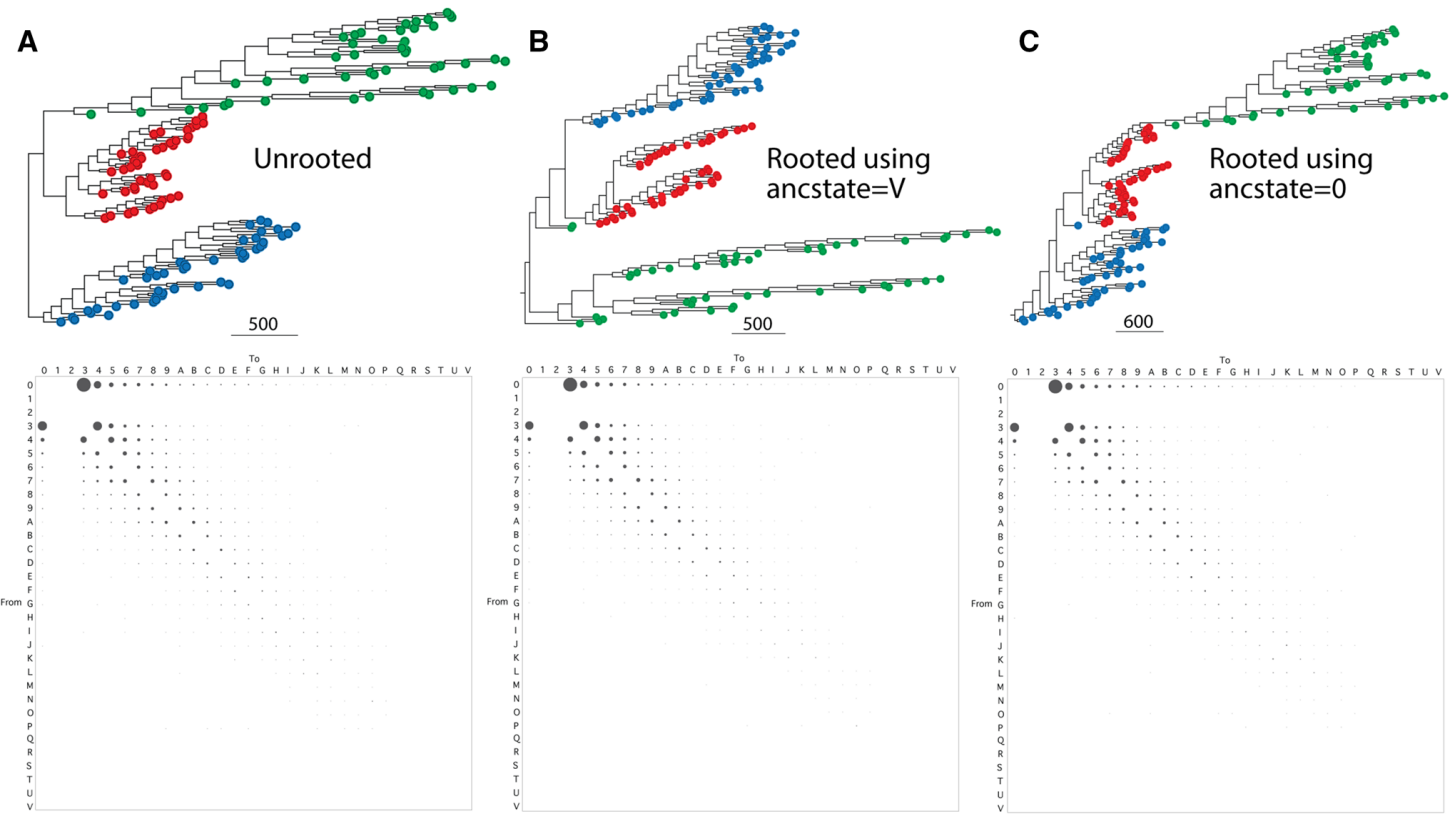
Deriving frequencies of character state changes from character state reconstructions. Phylogenetic trees were reconstructed from the *HK dataset* by treating coded domain abundance values as undirected unordered (Fitch) characters and producing unrooted trees (**a**), or trees rooted with Lundberg using ancstate = V (**b**) or ancstate = 0 (**c**). Bubble charts were derived from character state reconstruction of changes along the branches of the optimal trees. Taxa belonging to Archaea, Bacteria and Eukarya are labeled with red, blue and green circles, respectively. Tree statistics: **a** 2 optimal trees, tree length = 44,784, retention index = 0.499; **b** 4 trees, length = 45,872, retention index = 0.473; **c** 8 trees, length = 45,063, retention index = 0.495

Proteomic change in structural domain abundance has not been modeled for phylogenetic analysis. We, therefore, used the most agnostic model that is compatible with the HK model to generate unrooted trees (Fig. 3a) or trees rooted with Lundberg (Fig. 3b, c) from the *HK dataset* of Harish et al. (2013) and derived frequencies of character change by character state reconstruction. These frequencies can be used to build transformation model types that best match phylogenetic data. As mentioned earlier, the agnostic undirected unordered characters of the Fitch type with 32 character steps serve as foundational basis of the HK model. Figure 3 shows bubble charts that describe relative frequencies of reconstructed character change for unrooted and rooted versions of the most parsimonious trees. In all cases, changes occurred most frequently in single steps. More importantly, gains were favored over losses throughout the stepmatrix of character state transformations. Most notable trends of increased frequencies of gains were evident in changes from state 0 to states 3–9 relative to their loss counterparts, changes from states 3–9 to 0. These patterns of change go counter to those of the HK model, a simple observation that challenges the claim that the HK model is ‘empirical’.

**Fig. 4:**
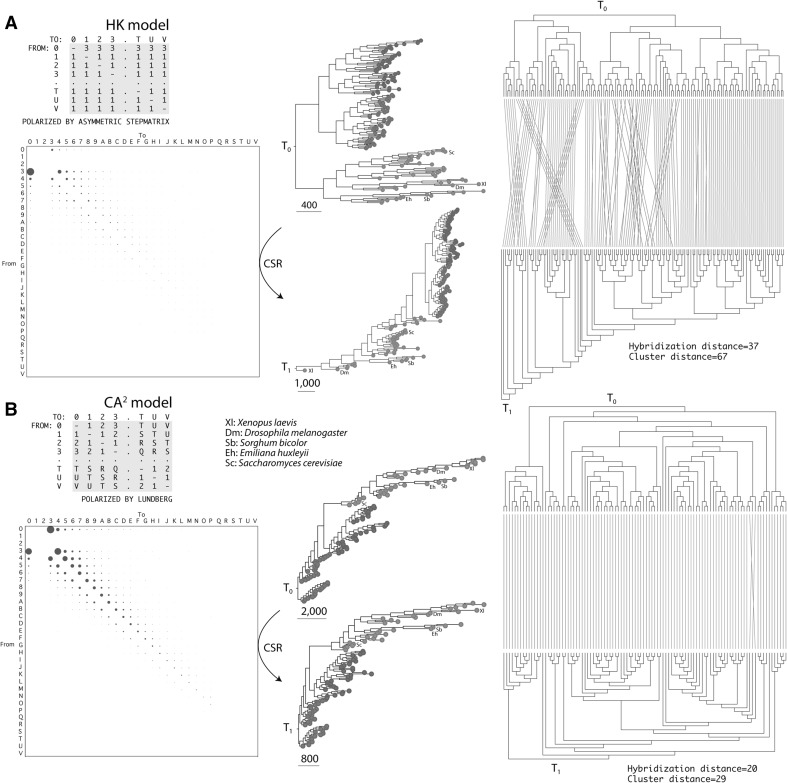
Testing the performance of stepmatrices of transformation costs derived from character state reconstruction (CSR) along the branches of trees (*T*_0_) obtained using the HK model (**a**) or the CA^2^ model (**b**). Significant changes in tree topology, revealed in tanglegrams comparing the two trees (right), were only observed when the refined tree (*T*_1_) obtained from the stepmatrix character type of the HK model was compared to the original tree (*T*_0_) in a first round of possible iterations. Higher hybridization and cluster distances indicate higher departures in tree topologies. Taxa belonging to Archaea, Bacteria and Eukarya are labeled with red, blue and green circles in connectors, respectively. Tree statistics for panel **a***T*_0_, 1 optimal tree, length = 53,746; *T*_1_, 2 trees, length = 81,966. Tree statistics for panel **b***T*_0_, 1 tree, length = 70,095, retention index = 0.723; *T*_1_, 1 tree, length = 54,906

Stepmatrices obtained from relative frequencies of character change can be applied to the characters of that the same dataset to build new ‘refined’ trees in iterative manner (Mickevich 1982). The idea encompasses the ‘dynamic weighting’ approach of Williams and Fitch (1990) that simultaneously and iteratively derives probabilistic models and trees until estimates and tree topologies are stabilized. We used this approach to compare the HK and CA^2^ models and test if a single round of iteration provided refined trees with stable topologies. The goal was to evaluate the likelihood fit between the model and data. First, we used the HK model (with its asymmetric stepmatrix) and the *HK dataset* to build an intrinsically rooted ToL and derive a transformation model type from frequencies of character change (Fig. 4a). As expected, patterns of increased gains that are present throughout the frequency bubble charts of the HK model were only biased in those cells in which the HK model originally taxed gains three times more than losses for the first gain (first row versus first column). Thus, the HK model reverses a crucial natural trend that exists in phylogenetic data (see Fig. 3). Construction of transformation model types from the frequency matrices using transformation weight functions of Wheeler (1990) were then used to build a new refined tree, which we find did not maintain the topology of the tree used to build the HK-derived stepmatrix model. Instead, the operation brought organisms with large genomes (e.g. *Xenopus laevis*) to the base of the ToL changing its rooting from monophyletic to paraphyletic. This reveals an artefactual attraction of large proteomes towards the root of the trees. Furthermore, tanglegrams and tree (hybridization and cluster) distances quantified significant topological mismatches between the trees. These results were not dependent on the dataset that was used. A similar outcome was obtained when trees were reconstructed with transformation model types derived from the HK model but using the *CA*^*2*^*dataset* instead (tanglegrams are described in Supplementary Fig. 1). Second, we used the CA^2^ model and the *CA*^*2*^*dataset* to build a ToL rooted with the Lundberg method. We then derived a transformation type from the frequency diagrams (which very much resembled those from unordered characters, Fig. 2). The tree built from this transformation type had a topology that was almost identical to the original tree, including the paraphyletic rooting in Archaea, as shown by tanglegrams and tree distances (Fig. 4b). A similar outcome was obtained when using the *HK dataset* but with increased tree distance values (Supplementary Fig. 1). Topology mismatches were expected given that the *HK dataset* included proteomes from obligate parasites shown to act as rogue data destabilizing leaves in phylogenetic analyses (Nasir et al. 2017). Table 1 describes hybridization and cluster distances of tanglegrams for the four sets of iterative reconstructions. Lower distance values imply better match between the initial and refined tree (less crisscross when comparing topologies), which in our analyses quantifies the fit between phylogenetic data and evolutionary model (Fig. 4). Our study reveals significant data-model mismatch when using the HK methodology, but not when the CA^2^ model was used.

**Table 1:** Tree distances of tanglegrams comparing phylogenetic trees reconstructed using the HK and CA^2^ models and transformation types that are function of frequency of chances derived from the respective models using CSR

		HK dataset	*CA*^2^ dataset
Hybridization distance	HK model	37	31
	CA^2^ model	29	20
Hardwired cluster distance	HK model	67	53
	CA^2^ model	48	29

Comparative analyses used either the *HK* or *CA*^*2*^ datasets

### Innovation and Innovation Spread are Different Concepts

When building ToLs from proteome data, characters describe the spread of individual structural innovations in the proteomes of the living world. They do not describe their rise, i.e. their first appearance in evolution, especially because taxa are not protein structural domains but proteomes. Character state models apply to each individual character, i.e., the proteomic occurrence or abundance of a FSF superfamily describing a ‘specific’ structural domain. Thus, each model relates to a single domain and describes change within a vector of FSF occurrences or abundances of that domain for a collective of sampled proteomes (not a vector of proteomes for each FSF). Characters, therefore, overwhelmingly describe how that FSF spreads in the lineages of the ToL.

Harish and Kurland (2017a) state: “The observed low frequencies of novel superfamily innovation as well as the higher frequencies of duplication and loss are summarized in the [HK] evolution model as lower propensities of innovation ... Since a lower relative propensity implies a higher constraint on the possible character-state transitions due to innovation, these transitions were assigned higher costs (penalty)”. This reasoning is faulty because the issue is not innovation but rather the balance of gains and losses in the trees of a domain that had already appeared in evolution. In fact, a structural innovation implies evolutionary transitions in protein fold space capable of generating a novel previously unseen structure. While the first appearance of these innovations are rare events, their retention and loss in trees of these highly conserved features is a durable and relatively common phenomenon. We have made the distinction between innovation and innovation spread explicit in a dynamic model of proteome evolution, which we found explains the phylogenomics of the CA^2^ characters (Tal et al. 2016). The model uses global birth–death differential equations and domain abundances as state values and is governed by two parameter classes, *λ*_*j*_ or the rate (birth minus death) of generating new variants of a same domain structure *j*, and *a*_*ij*_ or the rate of ‘forward’ transition from structure *j* to structure *i*. Individual FSFs under this model diversify in approximately exponential manner accumulating in a growing funnel with rate *λ*_*j*_. At some point, given rate *a*_*ij*_, the growing funnel generates a new FSF structure. This new structure is a unique and rare innovation (since *a*_*ij*_ < < *λ*_*j*_) that creates a new growing funnel of variants. The growth of protein domains in evolution is thus governed by sets of two parameter classes that are analogous to imitation and innovation parameters *q* and *p*, respectively, of the Bass innovation model (Norton and Bass 1987). Only *λ*_*j*_ contribute significantly to the spread of FSF innovations in ToLs, challenging the construction rationale of the HK model.

### Parametric Contingencies

The fully-reversible and non-reversible models we compare are stationary and time-reversible, i.e., the evolutionary processes responsible for character state change and their stochastic fluctuations have occurred long enough to make them independent of the starting character state and insensitive to both the vagaries of stochasticity and the flow of time. Thus, stepmatrices of transformation costs differ from tables of instantaneous transition rates (*Q* matrices) used by parametric model-based methods of phylogeny reconstruction, such as maximum likelihood or Bayesian analysis, which often use non-equilibrium transition probabilities to drive nonstationary directional Markovian (memory-less) evolution (e.g. Klopfstein et al. 2015). Instead, the stepmatrix models we compare are typical of discrete characters of the morphological type that are widely used in systematics and cladistics. It could be argued, however, that stepmatrix models could be tested and improved by a mathematical approximation to more realistic statistical models, using, for example, fitted model and time parameters inferred from an evolutionary model of proteome evolution (e.g. a dynamic birth–death model; Tal et al. 2016) or probabilities informed by stepmatrices obtained from CSR. Indeed, the HK model has been parametrized for Bayesian reconstruction from binary characters of FSF occurrence (Harish and Kurland 2017b) using the nonstationary model developed by Klopfstein et al. (2015). However, the validity of priors, the performance of the directional evolution model, and its statistical consistency have not been established or confirmed by simulation. Goloboff et al. (2018) highlight the perils of studying the evolution of morphological features with a model originally developed for restriction sites of nucleic acid sequence. In the case of HK, these morphological features take the form of high-level protein structural topologies, the evolution of which is poorly understood and is far away from the highly dynamic changes that often bring molecular sequences to saturation. In fact, Goloboff et al. (2018) discourage the use of the parametric model-based approach of reconstruction altogether by showing that parsimony performs as well or better than model-based methods for discrete morphological characters of the type proposed by Klopfstein et al. (2015). Instead, and despite the unsubstantiated belief that parametric models are superior, a parsimony “common sense” approach to what is known or not known of the evolutionary process shields against von Neumann’s fitting elephants—*“*With four parameters I can fit an elephant, and with five I can make him wiggle his trunk” (quoted by Enrico Fermi via Freeman Dyson). It also avoids taking a ‘philosophical’ stance with stochastic models of change. In this regard, the histories of complicated traits such as fold structures are likely biological singularities unfolding at different levels of molecular organization, i.e. historical random walks in the highly structured and percolated neighborhoods of genotype space, which are tailored by the function and structure of macromolecules (e.g. Ferrada and Wagner 2010). Concerns about the statistical inconsistency of parsimony developed for molecular sequences do not apply to morphological features, vanish with larger datasets, and cannot be adequately validated by simulation, especially when the model used to evolve the data has not been independently validated or when it is not supported by empirical evidence (Goloboff et al. 2018). In turn, many assumptions of nucleic acid models do not apply to morphological-type features, including the use of: (i) not more than 4-character states; (ii) fixed substitution rates, (iii) equilibrium of compositional frequencies often linked to substitution rates; (iv) constant selection pressures through time; and (v) concerted increases or decreases of transition probabilities for all characters at the same branches. Similarly, many assumptions of morphological-type features are not considered in models of sequence evolution, including assumptions explaining gain-loss (the effect of indels or genomic rearrangements) or character state independence (the effect of molecular structure). This blurs the careful balance of parameters and realism that propels the justification of parametric evolutionary models.

### The Archaea-First Model and the Paraphyletic Rooting of the ToL

The basal branches of a ToL define different models of diversification of life. All rootings with the CA^2^ model and rootings with the CA^2^ and HK models refined by the ‘dynamic weighting’ approach were paraphyletic (Fig. 4). These paraphyletic rootings can be interpreted to result from early grades, “groups of diversifying organisms (primordial archaeons) in active transition that were initially unified by the same and archaic level of physiological complexity” (Caetano-Anollés et al. 2014). When obligate parasites were excluded (e.g. *CA*^*2*^*dataset*) to avoid the effect of rogue taxa (Nasir et al. 2017), phylogenies obtained with the better-fitting CA^2^ model placed archaeal organisms at their base, and therefore, supported once again the Archaea-first scenario for origins of diversified life (Caetano-Anollés et al. 2014). This scenario suggests that the ‘turning point’ of origin of grades during the late evolution of the common ancestor of life was triggered by a significant transition process capable of eliciting an evolutionary ‘crystallization’ (sensu Carl R. Woese). Remarkably, phylogenomic analysis provides strong support to archaeal organisms diversifying by an early reductive evolutionary force and later on by co-evolution with other emerging lineages of the ToL (Staley and Caetano-Anollés 2018).

## Conclusions

As stated by Farris (1983), *“*Science requires that choice among theories be decided by evidence, and the effect of an ad hoc hypothesis is precisely to dispose of an observation that otherwise would provide evidence against a theory. If such disposals were allowed freely, there could be no effective connection between theory and observation, and the concept of evidence would be meaningless”. Here, Farris refers to the need of minimizing ad hoc hypotheses of homoplasy when reconstructing history. Over decades, this rationale developed into modern phylogenetic analyses. Proposals of character state models for phylogenomic reconstruction must be empirically grounded and must minimize the number of ad hoc and auxiliary assumptions. Here, we evaluate the two models that have been used so far to build rooted ToLs from a census of structural domains in proteomes. We find that the standard use of ordered Wagner characters and Weston’s generality rooting criterion is superior to the use of unordered characters and asymmetric stepmatrices. The standard models match reconstructed frequencies of character change and are faithful to the distribution of serial homologies in trees. In contrast, the asymmetric stepmatrix models do not reflect the data. Instead, they attract organisms with large proteomes to the base of the rooted trees while violating the triangle inequality of distances. They also pose many other difficulties. For example, a 32-state stepmatrix parsimony model ventures into Kluge’s slippery slope of deciding to add or not 992 new ad hoc assumptions that can support a ‘pre-specified’ monophyletic tree topology, without paying attention to how the morphological-type biological characters might actually evolve (Goloboff et al. 2018). This highlights the aprioristic perils of disposing of countering evidence in natural history reconstruction.

## Electronic supplementary material

Below is the link to the electronic supplementary material.

Supplementary material 1 Supplementary Fig. 1. Tanglegrams with labeled taxa for the entire experimental set described in Table 1 and partially described in Fig. 4. (TIF 2857 KB)
