# Supplementary figures and images for: Testing Empirical Support for Evolutionary Models that Root the Tree of Life
Source: J Mol Evol. 2019 Mar 18;87(2):131–42. doi: 10.1007/s00239-019-09891-7 (PMC6443624; doi:10.1007/s00239-019-09891-7)

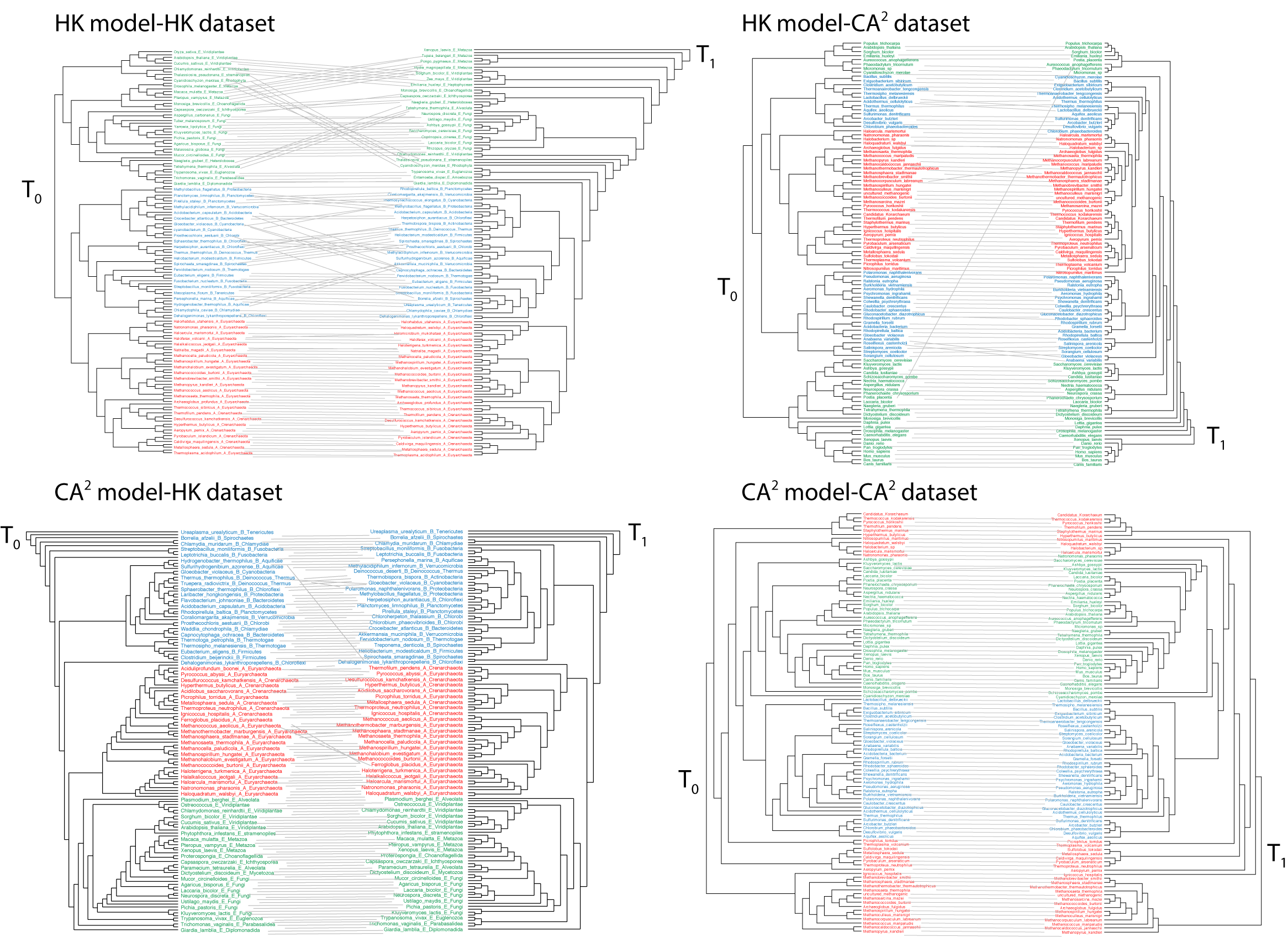

Supplement: Supplementary file 1 — Supplementary material 1 Supplementary Fig. 1. Tanglegrams with labeled taxa for the entire experimental set described in Table 1 and partially described in Fig. 4. (TIF 2857 KB) [file 239_2019_9891_MOESM1_ESM.tif]
